# ShinyKGode: an interactive application for ODE parameter inference using gradient matching

**DOI:** 10.1093/bioinformatics/bty089

**Published:** 2018-02-27

**Authors:** Joe Wandy, Mu Niu, Diana Giurghita, Rónán Daly, Simon Rogers, Dirk Husmeier

**Affiliations:** 1 Glasgow Polyomics, University of Glasgow, Glasgow, UK; 2 School of Computing, Electronics and Mathematics, University of Plymouth, Devon, UK; 3 School of Mathematics and Statistics, University of Glasgow, Glasgow, UK; 4 School of Computing Science, University of Glasgow, Glasgow, UK

## Abstract

**Motivation:**

Mathematical modelling based on ordinary differential equations (ODEs) is widely used to describe the dynamics of biological systems, particularly in systems and pathway biology. Often the kinetic parameters of these ODE systems are unknown and have to be inferred from the data. Approximate parameter inference methods based on gradient matching (which do not require performing computationally expensive numerical integration of the ODEs) have been getting popular in recent years, but many implementations are difficult to run without expert knowledge. Here, we introduce ShinyKGode, an interactive web application to perform fast parameter inference on ODEs using gradient matching.

**Results:**

ShinyKGode can be used to infer ODE parameters on simulated and observed data using gradient matching. Users can easily load their own models in Systems Biology Markup Language format, and a set of pre-defined ODE benchmark models are provided in the application. Inferred parameters are visualized alongside diagnostic plots to assess convergence.

**Availability and implementation:**

The R package for ShinyKGode can be installed through the Comprehensive R Archive Network (CRAN). Installation instructions, as well as tutorial videos and source code are available at https://joewandy.github.io/shinyKGode.

**Supplementary information:**

Supplementary data are available at *Bioinformatics* online.

## 1 Introduction

Mathematical modelling using ordinary differential equations (ODEs) is commonly used in systems and pathway biology. In this modelling paradigm, ODE parameters are often unknown and the aim of inference is to estimate parameters of the dynamical system. Generally this involves numerically solving the system of ODEs at each step of the inference procedure and evaluating how well the inferred parameters match the data. However, since non-linear ODEs typically have no closed-form solution, solving ODEs requires computationally expensive numerical integration. In recent years, approximate methods based on gradient matching, which allow estimating the unknown ODE parameters without the need for numerical integration, have been gaining popularity.

Gradient matching circumvents the numerical integration step by minimizing the difference in the derivatives of the interpolated data and the state variables predicted by the ODEs. The KGode package, available in the Comprehensive R Archive Network (CRAN), implements the gradient matching methods in Niu *et al.* (2016, 2017) to approximate ODE parameters. However, using the KGode package can be challenging for ordinary users as it requires expert knowledge in the R programming language. In this paper, we introduce an interactive application, *ShinyKGode*, built upon the KGode package, which provides a user-friendly interface to perform gradient matching and to visualize its results.

## 2 Methods and implementation

ShinyKGode is implemented on top of Shiny, a framework in R to develop interactive applications. Parameter estimation in our application relies on the KGode package, which implements fast parameter inference using gradient matching (Niu *et al.*, 2016, 2017). The SBMLR package is used for parsing of user-defined models in the Systems Biology Markup Language (SBML), while plotting is done through the ggplot package.

**Fig. 1.**
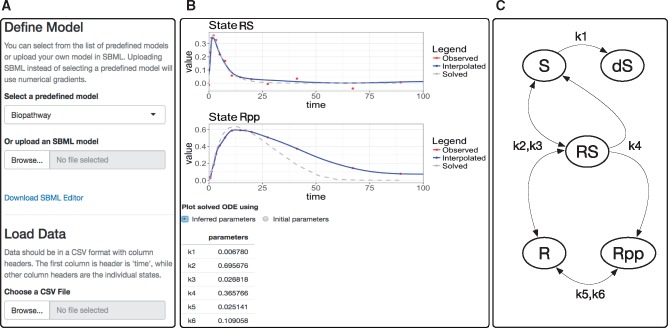
(A) The *Model* panel of ShinyKGode shows how users can load custom models in SBML, as well as select from the three benchmark models. Synthetic data can be generated, while observed data can be loaded. **(B)** Example results of gradient-matching based inference for two system states (*RS*, *Rpp*) and all kinetic parameters of the Biopathway (BP) model (Vyshemirsky and Girolami, 2008). This model, available as one of the benchmark models in ShinyKGode, describes the interaction of five protein isoforms, S,dS,R,RS,Rpp, in a signal transduction pathway through a combination of mass action and Michaelis–Menten kinetics having six parameters (labeled $k_{1:6}$). We generate noisy data by numerically solving the ODEs for $k_{1}=0.07,\;k_{2}=0.6,\;k_{3}=0.05,\;k_{4}=0.3,\;k_{5}=0.017,\;k_{6}=0.3,\;t\in (0,100)$ and initial conditions S(0)=1, dS(0)=0, R(0)=1, RS(0)=0 and Rpp(0)=0. This produces 14 data points, which are used as data for gradient matching with warping. Inference takes approximately 2 min on our computer (2.6 GHz Intel Core i7). Plots of the observed data alongside the interpolated signals and the solutions of the ODEs for the inferred parameters are shown for each system state. A table of inferred parameters can also be downloaded. **(C)** The network diagram of the Biopathway model

Running the application will open the *Model* panel to select or upload dynamical models and provide data for inference (Fig. 1A). Three standard benchmark models (Lotka-Volterra, Fitz-Hugh-Nagumo and the Biopathway models) are available for illustration, and users can easily load their own SBML model into the application. To provide data, users can generate synthetic data or upload their own data. Data should be specified in a Comma-Separated Value (CSV) format having column headers, where the first column header is called ‘time’, and other column headers are labels for the individual system states. Each row in the CSV file contains the observed values of time and states (for more details, see Supplementary Section S1).

Once the model and data are specified, parameter estimation can be performed from the *Inference* tab. The framework of Reproducing Kernel Hilbert Spaces (RKHS) is used for fitting the gradient in KGode, and various inference options (including the choice of kernel function in RKHS and the initial values for optimization) can be adjusted from this tab. The Radial Basis Function and Multi-Layer Perceptron kernels are provided in ShinyKGode. Clicking the *Infer* button will run gradient matching, and a progress bar is shown. From the *Diagnostic* tab, users can also see plots for assessing convergence of the optimization algorithm used for inference. As an option, we also provide a parametric bootstrapping method to estimate parameter uncertainty (Supplementary Section S2). Methodological details are provided in Niu *et al.* (2016, 2017).

The KGode package also allows for ODE regularization and warped gradient matching functionalities described in Niu *et al.* (2016, 2017). These are advanced options that can lead to a significant improvement in parameter estimation accuracy. In ShinyKGode, both ODE regularization and warping are options that can be enabled. In Figure 1, we provide an example demonstrating how ShinyKGode can be used to perform parameter inference with warping in a simple protein signal transduction pathway, and on our website, we also provide example videos that demonstrate the benefits of enabling these advanced options.

## 3 Conclusion

In this application note, we have introduced ShinyKGode, an interactive application to perform parameter inference in biological dynamical systems using gradient matching. The application can easily be used by biologists who want to estimate parameters of mathematical models of biopathways based on ODEs from noisy data, and related applications.

## Funding

JW, MN, SR and DH were supported by EPSRC (EP/L020319/1). RD was supported by Wellcome (105614/Z/14/Z).

*Conflict of Interest*: none declared.

## Supplementary Material

Supplementary Data
