# Supplementary material for: ShinyKGode: an interactive application for ODE parameter inference using gradient matching
Source: Bioinformatics. 2018 Feb 27;34(13):2314–5. doi: 10.1093/bioinformatics/bty089 (PMC6022662; doi:10.1093/bioinformatics/bty089)
Supplement: Supplementary Data [file bty089_supplement.pdf]

ShinyKGode: an Interactive Application for ODE Parameter Inference Using Gradient Matching

Joe Wandy[a], Mu Niu[b], Diana Giurghita[c], Rónán Daly[a], Simon Rogers[d], and Dirk Husmeier[c,*]

[a]Glasgow Polyomics, University of Glasgow, United Kingdom
[b]School of Computing, Electronics and Mathematics, University of Plymouth, United Kingdom
[c]School of Mathematics and Statistics, University of Glasgow, United Kingdom
[d]School of Computing Science, University of Glasgow, Glasgow, United Kingdom

February 12, 2018

1 Supplementary Section S1: Data File Format

The required file format for the user-supplied input data is a Comma-Separated Value (CSV) file with a header line. This header line takes the form of:

```
time, state1, state2, state3, ...
```

The first column header must be called '*time*'. Other column headers must match the names of the system states defined in the model. Each row in the CSV file contains the observed values of time and states. For example, to provide data for a model with three system states $X1$, $X2$ and $X3$:

```
"time","X1","X2","X3"
0,0.999030827591603,-0.0133988956955102,0.946765420910203
0.626619079036199,0.687815675613841,0.00765294565525593,0.728723065168827
1.30771194878186,0.554927692129674,0.0858368493767004,0.575695896673811
```

2 Supplementary Section S2: Parametric bootstrapping to estimate the parameter estimation uncertainty

We have implemented the following parametric bootstrapping procedure to estimate the parameter estimation uncertainty. Take the interpolant obtained from the final parameter estimates, $q*$, and compute the residuals by comparison with the data. Let us call the set of residuals E.

We then generate an ensemble of hypothetical data, $D_1, \ldots, D_K$, by drawing residuals with replacement from E and adding these values to the interpolant. Repeat, for $D_1, \ldots, D_K$, the same inference procedure that was performed on the true data, each time initialising the optimisation with $q*$. This procedure returns a set of bootstrap estimates, $\{q_1, \ldots, q_K\}$, which is summarised by the median absolute deviation (MAD); this is a robust measure of variability that is less susceptible to outliers than the standard deviation. We multiply the MAD with a constant scale factor of $k = 1.4826$, which makes it a consistent estimator for the estimation of the standard deviation of a normally distributed random variable. When the warping option is combined with the bootstrap option, the time warping from the original data is kept fixed. We effectively treat the warping as a pre-processing step that precedes the actual parameter estimation and therefore only needs to be carried out once. While this simplification may lead to a small underestimation of the parameter uncertainty, we note that the time warping is the computational bottleneck of the numerical procedure, and fixing the warping thus provides the best trade-off between accurate uncertainty estimation and keeping the computational costs acceptably low.

The bootstrap procedure described above can be enabled through the ShinyKGode interface by toggling the 'Bootstrap' option to *on*. In the interface, the MAD computed from the boostrap analysis is shown under the *bootstrap uncertainty* column of the output table. The user can use a bootstrap sample size, where larger values give more accurate estimates at higher computational costs. The default value has been set to 12.
